# Serendipitous SAD Solution for DMSO-Soaked SOCS2-ElonginC-ElonginB Crystals Using Covalently Incorporated Dimethylarsenic: Insights into Substrate Receptor Conformational Flexibility in Cullin RING Ligases

**DOI:** 10.1371/journal.pone.0131218

**Published:** 2015-06-29

**Authors:** Morgan S. Gadd, Emil Bulatov, Alessio Ciulli

**Affiliations:** Division of Biological Chemistry and Drug Discovery, College of Life Sciences, University of Dundee, James Black Centre, Dow Street, Dundee, United Kingdom; Centro Nacional de Biotecnologia—CSIC, SPAIN

## Abstract

Suppressor of cytokine signalling 2 (SOCS2) is the substrate-binding component of a Cullin-RING E3 ubiquitin ligase (CRL) complex that targets phosphorylated hormone receptors for degradation by the ubiquitin-proteasome system. As a key regulator of the transcriptional response to growth signals, SOCS2 and its protein complex partners are potential targets for small molecule development. We found that crystals of SOCS2 in complex with its adaptor proteins, Elongin C and Elongin B, underwent a change in crystallographic parameters when treated with dimethyl sulfoxide during soaking experiments. To solve the phase problem for the new crystal form we identified the presence of arsenic atoms in the crystals, a result of covalent modification of cysteines by cacodylate, and successfully extracted anomalous signal from these atoms for experimental phasing. The resulting structure provides a means for solving future structures where the crystals must be treated with DMSO for ligand soaking approaches. Additionally, the conformational changes induced in this structure reveal flexibility within SOCS2 that match those postulated by previous molecular dynamics simulations. This conformational flexibility illustrates how SOCS2 can orient its substrates for successful ubiquitination by other elements of the CRL complex.

## Introduction

In structural biology and structure-based drug design the existence of successfully solved and modelled crystallographic protein structures is critical to further advancements. These structures are important to understand how a protein functions and guide the development of small molecules targeting it. Additionally, for novel homologous or ligand-bound structures, the model itself provides the necessary information to solve the phase problem using molecular replacement (MR) or isomorphous refinement with the model as a template. These approaches rely on the novel structure to be sufficiently similar or isomorphous, respectively, such that the template is an adequate estimate of phases to enable the phase problem to be solved by these methods. In our crystallographic work with a complex of suppressor of cytokine signalling 2 (SOCS2), Elongin C (EloC) and Elongin B (EloB) we have encountered such a difficulty in solving the phase problem.

SOCS proteins such as SOCS2 are negative regulators of cytokine signalling of the Janus kinase/signal transducer and activator of transcription (JAK-STAT) pathway [1]. The SOCS proteins each have a similar domain architecture. They contain a central SH2 domain that specifically recognizes phosphorylated tyrosine residues, a hallmark of cytokine signalling [2]. SOCS proteins also have a C-terminal SOCS box that comprises a BC box that recruits EloB and EloC (EloBC) [3–4] and a Cul5 box that recruits Cullin 5 (Cul5) [5]. All of the SOCS family proteins bind to EloBC using their SOCS box, and both of these are required to recruit Cul5 [6]. Cul5, EloB and EloC are common components of Cul5-based cullin-RING ligases (CRLs).

CRLs are multi-protein complexes that act as E3 ubiquitin ligases [7–8]. They recruit an E2 conjugating enzyme, Cdc34/Ube2r1, carrying ubiquitin and via a substrate-binding protein, such as SOCS2, selectively transfer the ubiquitin to their substrate targets [9]. Using its SH2 domain, SOCS2 interacts with a number of proteins, including growth hormone receptor (GHR), insulin-like growth factor-I receptor, erythropoietin receptor, leptin receptor and epidermal growth factor receptor, when specific tyrosine residues on these proteins are phosphorylated [10–14]. SOCS2 has been shown to bind GHR as a substrate for ubiquitination and thus drive its degradation by the proteasome [15]. Other receptors interacting with SOCS2 are likely to be regulated in the same manner. These targets indicate there may be key roles for SOCS2 in somatic growth, the central nervous system, metabolic regulation, innate immunity and cancer [16]. Thus therapeutic developments targeting the activity of SOCS2 may lead to beneficial outcomes in a range of physiological and pathophysiological processes.

We have identified and validated a number of small molecule binders to a SOCS2:EloC:EloB complex using biophysical approaches. We sought to elucidate the binding sites and binding modes of these small molecules using x-ray crystallography. Despite successfully producing crystals of the complex that were isomorphous to those reported previously (PDB: 2C9W) [17], we found that when soaked with dimethyl sulfoxide (DMSO) alone and DMSO solutions of the compounds these crystals undergo a change in spacegroup and unit cell dimensions. As described above, this change resulted in difficulties that prevented solving the phase problem using MR approaches. We thus sought an alternative means of obtaining phases for the structure of DMSO-treated crystals.

We identified the presence of arsenic atoms in the 2C9W crystal structure as a result of covalent modification of surface cysteine residues with cacodylate buffer. We report here the successful resolution of the phase problem for DMSO-treated SOCS2:EloC:EloB crystals using arsenic single-wavelength anomalous dispersion (As-SAD). The structure reveals significant conformational changes that have resulted from the DMSO treatment that caused the decreased crystal symmetry. These conformational changes illustrate the hinging motion between the two domains of SOCS2 (as well as other SOCS box and F-box containing proteins) that may help to accurately position substrates for ubiquitination by CRL complexes, consistent with previous suggestions based on molecular dynamics simulations.

## Results

**Fig 1:**
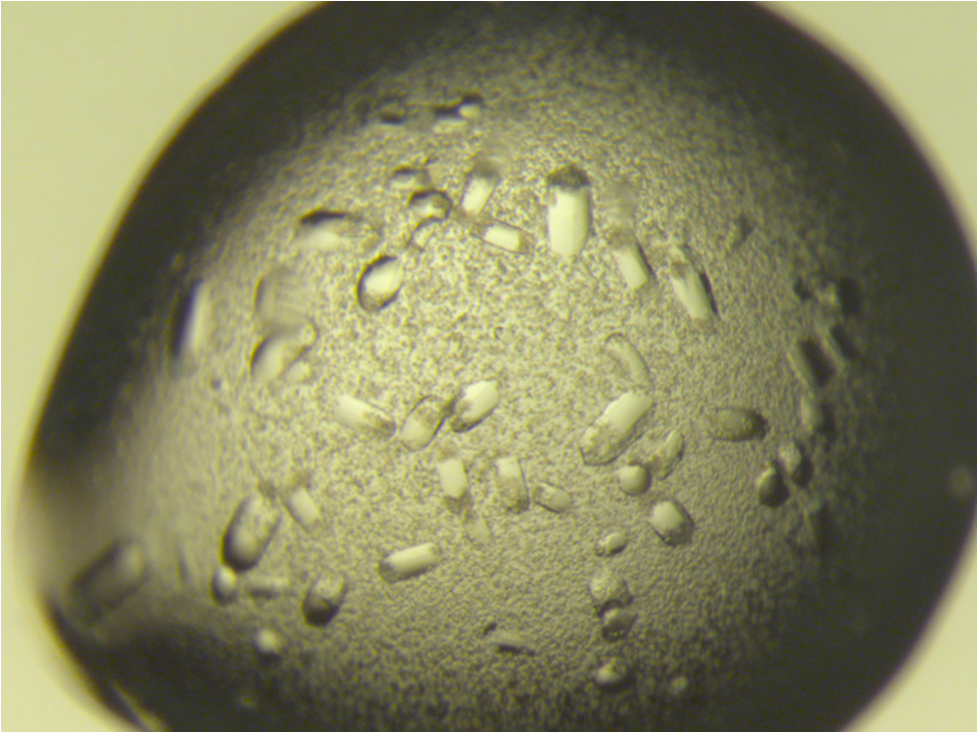
Crystals of the SOCS2:EloC:EloB complex grown after 7 d. The crystals shown here are 200–300 μm in the longest dimension.

In our crystallographic efforts on SOCS2:EloC:EloB we were unable to reproduce the crystallization condition previously reported by Bullock *et*. *al*. [17] containing 0.08 M sodium cacodylate (pH 6.5), 1.6 M ammonium sulfate and 0.16 M sodium chloride, with the protein complex in 50 mM Hepes, pH 7.5, 250 mM NaCl, 2.5% (v/v) glycerol and 10 mM dithiothreitol (DTT). Consequently, we attempted our own sparse-matrix screening to identify more readily reproducible crystallization conditions. Crystals were observed in Molecular Dimensions JCSG-*plus* condition 2.11 containing 0.08 M sodium cacodylate, pH 6.5, 0.16 M calcium acetate, 14.4% (w/v) PEG8000 and 20% (v/v) glycerol with the protein complex in 25 mM Hepes, pH 7.5, 250 mM NaCl and 10 mM DTT. Further refinement of the protein complex sample in this solution yielded single crystals that grow up to 500 μm in length in conditions of 0.08 M sodium cacodylate pH 7.2, 0.16 M calcium acetate, 14.5% (w/v) PEG3350 and 20% (v/v) glycerol (Fig 1).

Diffraction data were collected on a single crystal using Cu *K*α radiation as described. Indexing and merging of the data indicated the spacegroup to be *P*3_2_21 and the unit cell dimensions to be *a* = *b* = 105.5, *c* = 69.4, containing one instance of the SOCS2:EloC:EloB complex in the asymmetric unit (ASU). These indexing parameters are equivalent to those described previously [17]. The structure was solved to 2.6 Å resolution by MR using 2C9W as the search model and refined to an *R*_work_ of 23.6% and *R*_free_ of 30.4%. The resulting structure revealed no significant differences to that of 2C9W (RMSD = 0.40 Å; data not shown), indicating that both crystallization conditions yield crystals of the same form.

Having successfully produced SOCS2:EloC:EloB crystals we sought to utilize them to elucidate the structural details of small molecule binders identified during screening efforts (to be reported elsewhere). During these investigations we found that, having been soaked with the small molecule solutions in DMSO, the diffraction patterns of the crystals could no longer be indexed with the same space group and unit cell parameters as reported above. These data for DMSO-treated SOCS2:EloC:EloB crystals were instead successfully indexed with the alternative space group *P*3_2_ and approximate unit cell dimensions *a* = *b* = 186 Å, *c* = 67 Å. The change in unit cell dimensions and space group represents a six-fold increase in the size of the ASU, thus containing six SOCS2:EloC:EloB complexes instead of a single one.

To solve the phase problem for the new crystallographic parameters, diffraction data collected at resolutions from 3.3–3.8 Å were used in MR calculations (data not shown). Unfortunately a solution could not be found despite multiple attempts with a variety of different methods, including efforts to separately place SOCS2 and EloBC, as had been used to solve similar structures previously [17–20]. The difficulty of these MR calculations is most probably because of the six complexes (18 protein chains) to place in the rotation-translation function, but may be exacerbated by potential conformational changes that have driven this loss in crystallographic symmetry, the lower resolution of the diffraction data, as well as the fact that the complex is not a packed, globular unit.

**Fig 2:**
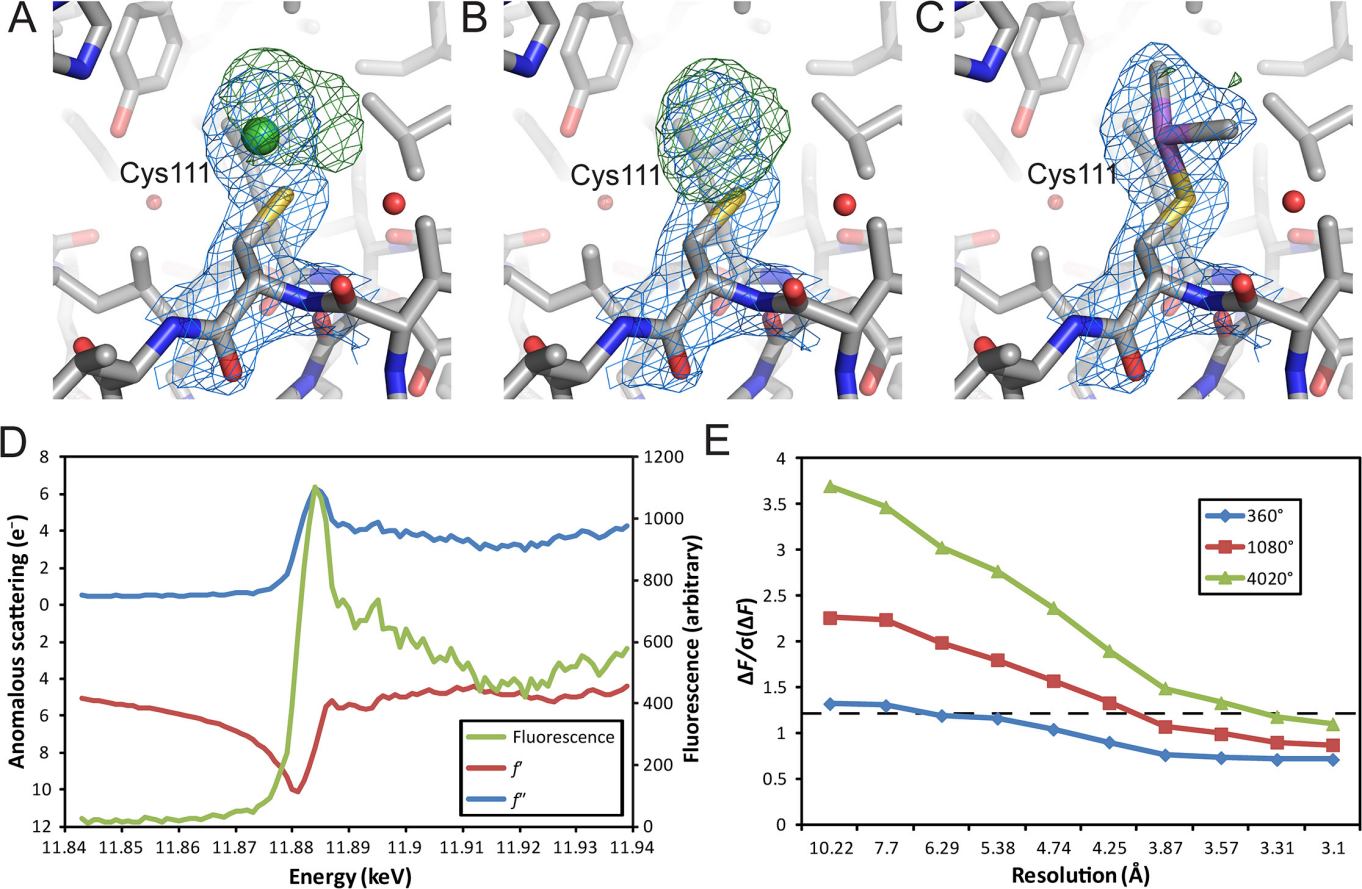
Identification of arsenic as a source of experimental phasing power. (A) The 2*F*_o_−*F*_c_ (*blue mesh*) and *F*_o_−*F*_c_ (*green mesh*) maps around modelled SOCS2_C111_ (*CPK colored sticks*) and adjacent Ni(II) (*green sphere*) from the PDB structure 2C9W. (B) As in A, with maps recalculated following removal of Ni(II). (C) As in A, with SOCS2_C111_ remodelled as dimethylarsenic cysteine. 2*F*_o_−*F*_c_ maps are contoured at 1σ and *F*_o_−*F*_c_ at 3σ. (D) A fluorescence scan (*green line*) and measurements of *f*≠ and *f*≡ (*red* and *blue lines*, respectively) identifying the presence of arsenic and resulting anomalous signals from DMSO-treated SOCS2:EloC:EloB crystals. (E) Observed anomalous signal [Δ*F*/σ(Δ*F*)] in diffraction data plotted against resolution for the As-Peak datasets collected. The cut-off of useful signal (1.2) is indicated by a dashed line [22].

In spite of these challenges we sought an alternative way to solve the phase problem for this structure. In the deposited model for 2C9W there are three Ni(II) ions modelled around the single complex in the ASU. Careful analysis of structure and electron density using the deposited structure factors for 2C9W suggested that the two Ni(II) ions modelled as being bound to cysteine residues (SOCS2_C111_ and EloB_C89_) inadequately satisfy the electron density in these parts of the structure (Fig 2A). Having removed the Ni(II) ions we identified that a covalent modification to the cysteine residues was a more likely explanation for the additional electron density (Fig 2B). By re-examining the crystallization and sample buffer conditions we determined that the presence of cacodylate and dithiothreitol (DTT) may have resulted in dimethylarsenic modifications to these thiol residues. Such a covalent modification was first observed and characterized by the reactivity of As(III)-thiolate intermediates (generated from DTT or β-mercaptoethanol) with the *Eco*RI methylase complex [21]. Following remodelling and refinement we observed that these modified sidechains fully satisfied the additional electron density at these cysteine residues (Fig 2C).

Arsenic sits adjacent to selenium in the periodic table and is a potential source of anomalous signal for experimental phasing. We performed a fluorescence scan to confirm the presence of arsenic and identified a wavelength of 11.8847 keV (λ = 1.04323 Å) for maximum production of *f*≠ and *f*≡ from the arsenic peak (Fig 2D). In an effort to conduct single-wavelength anomalous dispersion (SAD) phasing for the crystals we collected a series of datasets with increasing redundancy (Fig 2E). Due to the weakness of the observed anomalous signal, ultimately a dataset of 4020 degrees (redundancy ≈ 120) was used to ensure a SAD solution could be achieved (Table 1).

**Table 1:** X-ray diffraction data and model statistics for DMSO-treated SOCS2:EloC:EloB.

*Scaling statistics*	As-Peak	Native
Wavelength (Å)	1.04323	0.95372
Space group	*P*3_2_	*P*3_2_
Unit-cell parameters (Å)	*a* = *b* = 185.9, *c* = 67.3	*a* = *b* = 185.2, *c* = 67.2
Resolution limits (Å)	54.5–3.1 (3.27–3.10)	46.3–2.9 (2.98–2.90)
Completeness (%)	100 (100)	99.2 (100)
Wilson *B* factor (Å^2^)	64.2	59.6
Observed reflections	5659871 (744593)	219197 (17340)
Unique reflections	47227 (6906)	56676 (4449)
Redundancy	119.8 (107.8)	3.9 (3.9)
*R*_merge_ ^a^ (%)	12.0 (74.4)	6.7 (62.2)
*I*/*σ*(*I*)	52.6 (13.4)	15.6 (2.3)
CC_1/2_ (%)	100 (98.9)	99.9 (67.3)
*Phasing statistics*		
FOM_PHASER_	0.311	
FOM_RESOLVE_	0.310	
*Model refinement*		
*R*_work_ ^b^ (%)		22.9 (33.5)
*R*_free_ ^c^ (%)		28.7 (41.5)
No. of reflections used in refinement		53812 (4013)
No. of reflections in the test set		2862 (203)
Protein atoms		14871
Solvent molecules		33
RMSD bond length (Å)		0.006
RMSD bond angles (°)		1.01
Mean protein *B* factor (Å^2^)		77.5
*Ramachandran plot*, *residues in*		
Favored regions (%)		94.1
Allowed regions (%)		5.7
Disallowed regions (%)		0.2

Values in parentheses are for the highest resolution shell.

Values for the highest resolution shell are given in parentheses.

^a^ *R*_merge_ = Σ_*hkl*_ Σ_*i*_|(*I*_*i*_(*hkl*)–[*I*(*hkl*)]|/Σ_*hkl*_ Σ_*i*_ *I*_*i*_(*hkl*)

^b^ *R*_work_ = |*F*_obs_–*F*_calc_|/|*F*_obs_|, where *F*_obs_ and *F*_calc_ are the observed and calculated structure amplitudes, respectively.

^c^ *R*_free_ is *R*_work_ for the 5% validation set.

**Fig 3:**
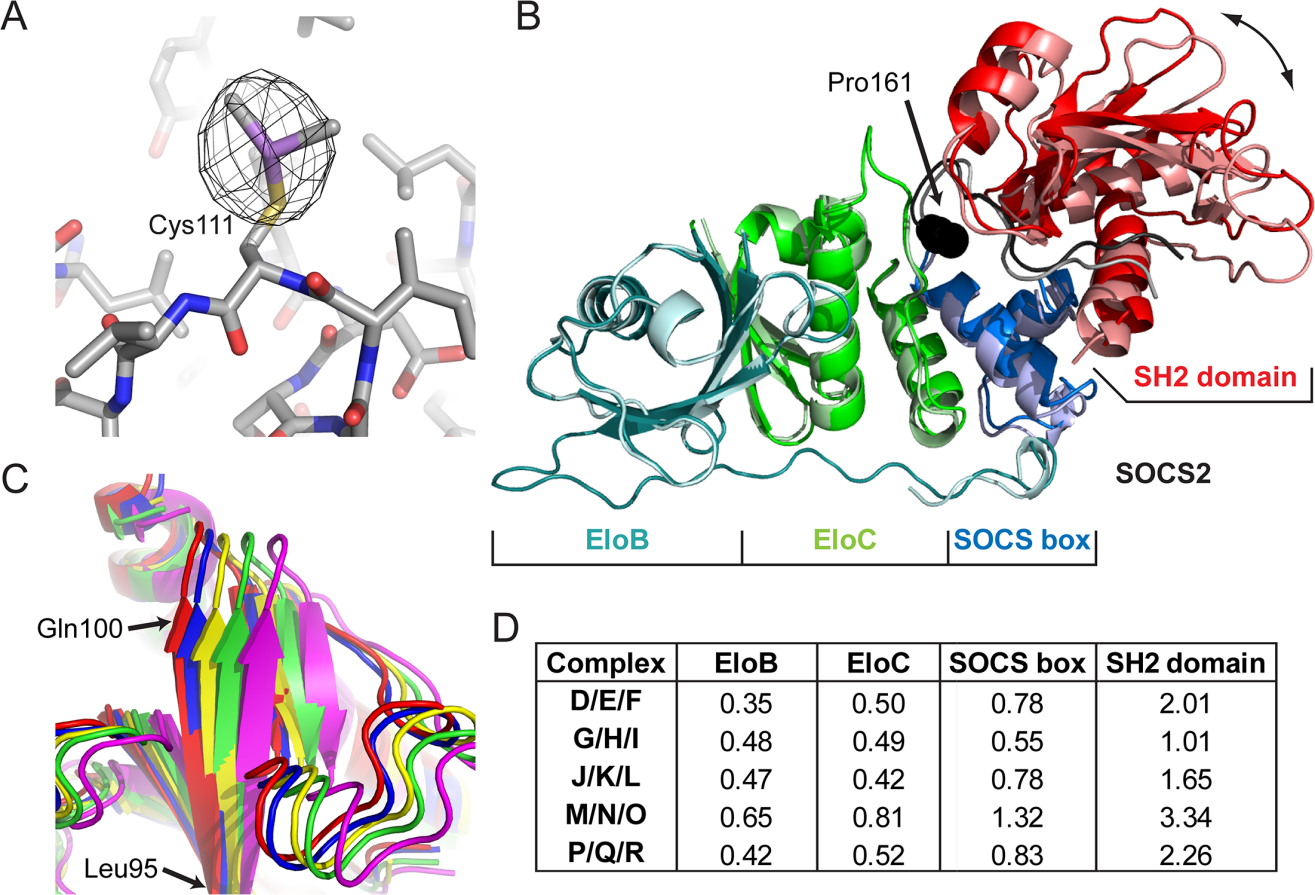
The structure of DMSO-treated SOCS2:EloC:EloB. (A) Anomalous difference map (*black mesh*) shown at 5σ calculated from As-Peak diffraction data in proximity to SOCS2_C111_ residue of Chain J (*CPK colored sticks*) with the dimethylarsenic modification. (B) Structure of the two most conformationally different complexes of DMSO-treated SOCS2-EloBC (A/B/C and M/N/O), aligned via the backbone atoms of EloB. Protein chains are shown as ribbons, with EloB (*cyan*), EloC (*green*), SOCS2 SOCS box (*blue*) and SOCS2 SH2 domain (*red*). Chains A/B/C are in darker colors and M/N/O lighter. The linker/hinging region between SOCS2 SOCS box and SH2 domain is shown in black/grey and residue Pro161 as spheres. (C) SH2 domains of DMSO-treated SOCS2-EloBC illustrating the five different conformations observed in the ASU. Chains A/B/C (*red*), D/E/F (*green*), G/H/I (*blue*), J/K/L (*yellow*) and M/N/O (*magenta*). Complexes have been aligned via the backbone atoms of EloB from each complex and superposed. Complex P/Q/R is not shown as its conformation is identical to that of D/E/F. (D) Root mean square deviation (RMSD) values (in Å) for the backbone atoms of EloB, EloC, SOCS2 SOCS-box domain (residues 162–198) and SOCS2 SH2 domain (32–134) when complexes are superposed as in *C*. RMSD values are between each complex and complex A/B/C.

Experimental phases for the structure of DMSO-treated SOCS2:EloC:EloB were determined by SAD using diffraction data collected at 11.8847 keV to 3.1 Å resolution. 12 individual arsenic peaks were found in the resulting experimental map. Automated model building produced a series of chains representing an incomplete, fragmented structure with an *R*_work_ of 37.9% and *R*_free_ of 43.4%. The experimental map and partial structure were used to align regions of the template structure 2C9W to place the six copies of the complex in the ASU. The 12 unique arsenic peaks were confirmed as sites of cacodylate modifications on cysteine residues (Fig 3A). Peaks for the six SOCS2_C111_ residues can still be observed in the anomalous difference map at approximately 20σ, whereas those for EloB_C89_ can only be observed at 5σ.

The six complexes in the asymmetric unit were refined against a native dataset processed to 2.9 Å resolution. The final model was refined to an *R*_work_ value of 22.9% and *R*_free_ of 28.7% (Table 1). Each complex in the model maintains the same overall structural assembly observed in 2C9W, however there are also differences. In EloB all residues but the initiating methionine are observed in the 2C9W structure. In our structure, however, residues 18 and 19 and six C-terminal residues of chain E, as well as residues 15–19, 32–37 and 78–96 of chain N are not visible in the electron density. In EloC residues 46–57 and 85–88 are disordered and not visible in the 2C9W structure, however the final loop (85–88) is traceable in the electron density within chains C, I and L of DMSO-treated SOCS2:EloC:EloB.

When aligned via the backbone atoms of the EloB proteins, significant deviations in the gross structure of the complex can be observed by analysing SOCS2 on the opposite side of the complex (Fig 3B). These deviations are well illustrated by residues 92–100 of SOCS2 when all complexes are aligned via the backbone atoms of EloB (Fig 3C). When analysing each component of the DMSO-treated SOCS2:EloC:EloB structure in this alignment we find that EloB, EloC and the SOCS2 box of SOCS2 are well aligned (average RMSD of backbone atoms = 0.62 ± 0.25), whereas significant deviations are observed for the SH2 domain of SOCS2 (average RMSD of backbone atoms = 2.1 ± 0.9) (Fig 3D).

## Discussion

Experimental phasing is a key element of crystal structure resolution, not only because it can provide a solution to the phase problem where MR proves insufficient, but also it eliminates phase bias in any successful MR solution. Sources of anomalous signal in native crystals include sulfur or phosphorous atoms, co-ordinated metal ions or unnatural amino acids, such as selenomethionine. Each of these approaches has drawbacks, for example sulfur-SAD requires precisely measured reflections to a reasonable resolution [23], metal ions are restricted to those proteins that bind them such as metalloproteins, and selenomethionine incorporation is not always practical. In this case the phasing atom, arsenic, has been incorporated as an essential step in the crystallization process, and thus the native crystals facilitate anomalous phasing.

**Fig 4:**
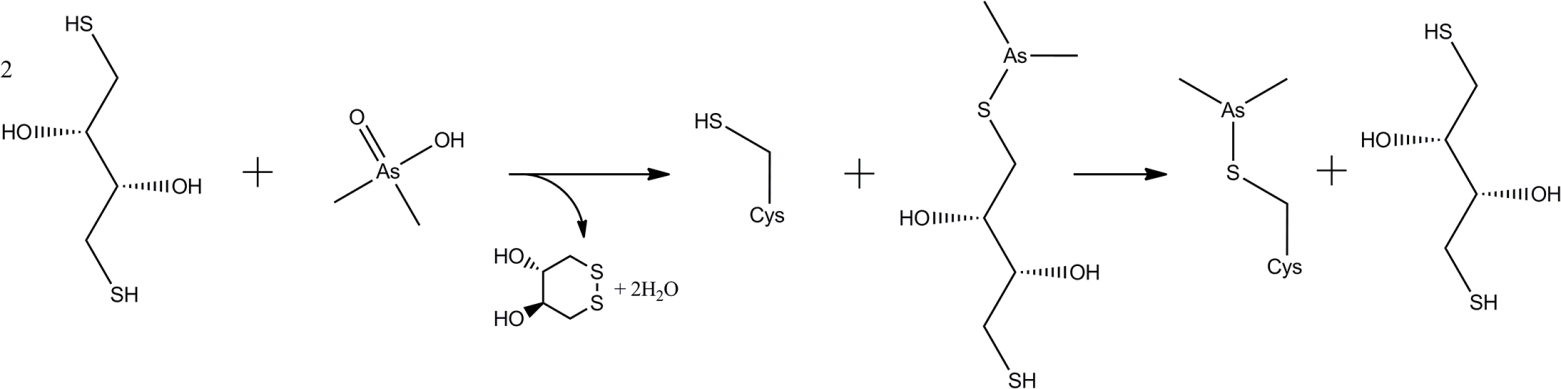
Mechanism for dimethylarsenic modification of cysteine residues proposed by Maignan *et al*. [29]. DTT reduces As(V) of cacodylate in its acid form (p*K*_a_ = 6.3) into an As(III)-containing dimethylarsenic-dithiothreitol conjugate. This adduct then reacts with a reduced cysteine sidechain to result in dimethylarsenic cysteine and a replenished molecule of DTT.

Arsenic is relatively underused as a potential source of anomalous signal for phasing. To date only five structures have been solved using the atom as a source of anomalous signal and deposited in the PDB, four of which were a product of cacodylate incorporation [24–27]. Compared to isomorphous replacement methods that soak heavy atoms, this approach results in only minor changes to the protein surface and happens during the crystallization process, and thus is more likely to facilitate crystal growth rather than inhibit it or disrupt grown crystals. Indeed, in our experience both SOCS2:EloC:EloB and a similar complex where SOCS2 is substituted by the von Hippel–Lindau tumor suppressor (pVHL) require both DTT and cacodylate to be present in the crystallization experiment for successful crystal growth [28]. For the crystallization of the catalytic domain of HIV-1 integrase the presence of both DTT and cacodylate was also observed to be an absolute requirement, and a mechanism by which the reaction took place was proposed (Fig 4) [29].

In the structure presented here the modified residue SOCS2_C111_ has the equivalent or lower thermal displacement (*B*) factors on the arsenic and methyl atoms than other atoms in the sidechains from the six complexes. On the other hand EloB_C89_ has significantly higher *B* factors for the arsenic and methyl atoms. This likely corresponds with the observation that SOCS2_C111_ contacts a surface formed by residues 87–91 of an adjacent SOCS2 molecule on a two-fold symmetry axis and is observable at 20σ in the anomalous difference map, whereas EloB_C89_ forms no similar crystal contacts and the arsenic atom is observable only at 5σ. SOCS2_C111_ therefore forms a highly ordered contact that may explain the beneficial effect of the dimethylarsenic modifications to the growth of these crystals. Thus the modification with cacodylate essentially generates a new sample of the same protein with a different potential for the formation of crystal contacts and thus successful crystallization.

To date 159 structures in the Protein Data Bank contain dimethylarsenic cysteine or dimethylarsinoyl cysteine (the two modifications that can result from cacodylate incorporation) as modelled residues. The usefulness of this buffer component for both potential phasing purposes and facilitating crystallization would suggest that it is extremely underutilized. Anecdotally this would appear to be because of the toxicity of cacodylate due to its reactivity with thiols [21, 30]. However, cacodylate is a common buffer component in the more popular sparse-matrix crystallization screens, and thus will continue to be identified as a potential crystallization hit [31–32].

Our structure of DMSO-treated SOCS2:EloC:EloB illustrates important features of conformational dynamics within this important regulatory complex. Differences between the individual complexes in the ASU of the structure are well illustrated when a superposition is performed via the backbones atoms of the EloB molecules (Fig 3B, 3C and 3D). In all cases the chains of EloB, EloC and the SOCS-box domain of SOCS2 are well aligned, with low RMSD values, indicating these parts of the complex form a rigid unit in relation to one another. In contrast the range of RMSD values for the SH2 domain of SOCS2 indicates a significant degree of conformational freedom between this domain and the remainder of the complex.

Molecular dynamics simulations have suggested that the highly conserved Pro161 residue of SOCS2, which lies just before the SOCS box (162–198), is a key component in imparting conformational flexibility at this hinge region (Fig 3B) [33]. The ensemble of models found in the DMSO-treated SOCS2:EloC:EloB structure reinforces this observation with the substrate-binding SH2 domain of SOCS2 alone exhibiting a clear hinging motion relative to the remainder of the complex (Fig 3B and 3C), consistent with the computational predictions [33]. A similar effect is observed when comparing the crystal structures of SOCS2:EloC:EloB when bound and not bound to Cul5 [17, 34]. However, the observed flexion between the SOCS box and SH2 domain in the Cul5-bound structure lies outside the range observed in the ensemble of the DMSO-treated SOCS2:EloC:EloB structure.

The computationally observed hinging motion is replicated in other SOCS-box and F-box substrate-binding proteins, such as pVHL and S phase kinase-associated protein 2 (Skp2), and appears to be regulated by the binding of substrate and/or adaptor proteins [33, 35]. A similar analysis to that above with published crystal structures of these proteins reveals similar effects. For the pVHL:EloC:EloB complex the hinging motion of the substrate-binding domain ranges with motion at one end for the pVHL:EloC:EloB:HIF-1α complex [36–37], then the four protomers of the pVHL:EloC:EloB apo complex [38] and at the other extreme the pVHL:EloC:EloB:Cul2 structure [39]. All seven of these structures show a similarly rigid alignment of EloB, EloC and the SOCS box of pVHL. When aligned via Skp1, the published structures of F-box containing proteins Skp2 and cell division control protein 4 (Cdc4) reveal the same rigidity between Skp1 and the F-box domain, but relative hinging motion for the leucine-rich repeat and WD40 repeat like domains in Skp1 and Cdc4, respectively [40–42]. As a common feature in SOCS-box and F-box containing proteins, the hinging motion and its regulation by substrate- and adaptor-protein binding is thus postulated to be critical for ensuring accurate positioning and orientation of the target substrate [33, 35]. These motions tie into global motions identified in CRL complexes that bring the substrate into proximity for ubiquitination by Cdc34 at the other end of the CRL complex [43].

The observed hinging motion between the SH2 domain and the remainder of the complex is likely to be a key driver of the change in crystal form from 2C9W to the structure presented here, as each complex (except for D/E/F and P/Q/R) has a slightly different orientation of the SH2 domain. The degree of disorder at unstructured loops in each complex differs (e.g. chain N lacks a total of 28 residues found in every other chain of EloB) and is likely to be another factor in the loss of crystallographic symmetry within the crystals. Due to these rather significant conformational changes the structure was not solvable using the data initially available to us (lower than 3.3 Å resolution) by MR using 2C9W or its individual components as search models. Experimental phasing using the arsenic atoms of the dimethylarsenic modifications was critical in providing a solution to the phase problem for this structure. While we were eventually successful in generating a MR solution from our subsequently collected data to 2.9 Å resolution, this structure revealed a more limited range of conformations which do not reflect the extent of hinging motions observed in our experimentally phased structure, suggesting phase bias in the former.

When protein crystals are soaked in solutions with molecules not found in the crystallization experiment they will sometimes be damaged or destroyed altogether, potentially cracking, losing order or resolubilising, particularly in the presence of organic solvents [44]. In a case such as this the disruption of the crystal lattice has been limited enough to maintain useful crystallographic diffraction, albeit with reduced crystallographic symmetry within the crystal. Similar effects to crystal properties have been observed previously. In the literature we identified one case where the addition of new molecules (NaBr) in a soaking condition for a peroxiredoxin resulted in a decrease in crystallographic symmetry, from *I*4 to *P*4_2_ [45]. On the other hand three cases of increased symmetry were identified: for crystals of the DNA mismatch repair protein MutS the presence of MnCl_2_ in the soaking solution results in an increase in symmetry from *P*2_1_ to *P*2_1_2_1_2_1_ and increased resolution of diffraction [46]; soaking of glutathione *S*-transferase crystals with zinc protoporphyrin resulted in a change of spacegroup from *P*2_1_2_1_2_1_ to *C*222_1_ [47]; and soaking again with NaBr on crystals of a pepstatin-insensitive carboxyl proteinase resulted in a change of symmetry from *P*2_1_2_1_2_1_ to *P*6_1_22 [48]. In all cases these soaking atoms were not visible in the electron density map (although in the latter case Br atoms were weakly visible in an anomalous difference map), suggesting that their influence on the physical properties of the soaking conditions was likely to be driving the observed alterations in crystal symmetry.

When crystal-soaking conditions alter but do not destroy the crystal properties and thus resultant x-ray diffraction, it is important to salvage such cases where possible. While typically a pre-existing solved structure should enable the phase problem to be addressed using MR, this was not the case here. The change in space group and unit cell dimensions in the presence of DMSO have resulted in a large increase in the number of protein molecules, as well as general disordering of the complex, making it very difficult to establish an MR solution with any ease. Thus, this structure provides a key avenue for the solution of ligand-bound structures of SOCS2:EloC:EloB where DMSO (and potentially other solvents) is required to solubilize ligands for successful soaking experiments. In turn these ligand-bound structures will be critical for the future development of chemical tools or potential small molecule therapeutics that modulate SOCS2-based CRL complexes.

## Materials and Methods

### Protein expression, purification and crystallization

SOCS2:EloC:EloB was expressed and purified as described previously [49]. Briefly, the complex was co-expressed in *Escherichia coli* BL21(DE3) from the pLIC (His_6_-SOCS2) and pCDF (EloBC) plasmids. The sequences are: human SOCS2 (residues 32–198; a gift from A. Bullock, Structural Genomics Consortium, Oxford, UK); EloB (amino acids 1–104); and EloC (amino acids 17–112). Protein expression was induced with isopropyl β-d-1-thiogalactopyranoside at 18°C for 12 h. SOCS2:EloC:EloB was extracted from lysed cells in the soluble fraction and purified by affinity chromatography using a HisTrap column (GE Healthcare). The His_6_-tag fused to SOCS2 was removed by cleavage with tobacco etch virus protease and the complex reapplied to the HisTrap column as a non-binding species. Finally the complex was purified by size-exclusion chromatography on a Superdex 75 16/600 column (GE Healthcare) in a buffer of 25 mM HEPES, pH 7.5, 250 mM NaCl and 10 mM DTT.

Crystallization conditions for SOCS2:EloC:EloB were identified using commercially available sparse-matrix screens (Qiagen and Molecular Dimensions). A 96-channel Phoenix high-speed liquid handling system (Art Robbins Instruments) was used to set up crystallization experiments using the sitting-drop vapor-diffusion method at room temperature. Crystallization drops were set up in ratios of 1:1 or 1:2 protein:liquor, respectively, and incubated at room temperature. Crystal formation and growth were monitored manually or by automated imaging using a Rock Imager 1000 (Formulatrix). Optimization of crystallization conditions was performed manually in 2-μl drops using the hanging- and sitting-drop vapor-diffusion methods at room temperature.

### X-ray diffraction data collection

SOCS2:EloC:EloB crystals were flash-frozen in liquid nitrogen without cryoprotection. For determining isomorphism with 2C9W, diffraction data were recorded on a Saturn 944HG+ CCD detector using x-rays produced by a Rigaku M007HF generator (Cu *K*α). To obtain anomalous dispersion data, DMSO-treated SOCS2:EloC:EloB crystals were also flash-frozen without cryoprotection. Diffraction data were collected on BM14 at the European Synchrotron Radiation Facility at 100 K on a single crystal. A fluorescence scan was performed to identify the presence of arsenic atoms and determine the wavelength for maximum dispersive effects. The As-Peak dataset was collected at a wavelength of 1.04323 Å for 4020 frames with an oscillation angle of 1° per frame. The native dataset was collected at a wavelength of 0.95372 Å for 120 frames with an oscillation angle of 1° per frame.

### Experimental phasing, model building and refinement

Diffraction data were indexed and integrated using Mosflm [50] and scaled and merged with Aimless in CCP4 [51–52]. The structure solution pipeline AutoSol in the PHENIX software suite [53–54] was used to establish experimental phases by identifying arsenic atom positions and generate initial phases (PHASER) [55], then to perform density modification, solvent flattening and automated model building into the resulting map (RESOLVE) [56]. The incomplete model generated by RESOLVE was completed with the aid of the previously published SOCS2:EloC:EloB structure by a series of alignments and manual building in Coot [57]. The resulting structure was refined with REFMAC5 [58] using isotropic temperature factors, TLS groups and NCS restraints. The MOLPROBITY server [59] was used to validate the structure by identifying steric clashes and geometric problems. The coordinates and structure factors of DMSO-treated SOCS2:EloC:EloB have been deposited in the Protein Data Bank with the PDB code 5BO4.
